# Supplementary material for: EcoLiDAR: An economical LiDAR scanner for ecological research
Source: PLoS One. 2024 Jun 25;19(6):e0298712. doi: 10.1371/journal.pone.0298712 (PMC11198765; doi:10.1371/journal.pone.0298712)
Supplement: S1 File — Additional Information regarding the EcoLiDAR software, building and operation. (DOCX) [file pone.0298712.s001.docx]

Title: EcoLiDAR: an Economical LiDAR scanner for Ecological research

**Supplementary material**

**Fig 1**. Pan-axis gear system. a) stepper motor; b) 20-teeth timing pulley; c) timing belt; d)-60 teeth timing pulley; e) slip ring.


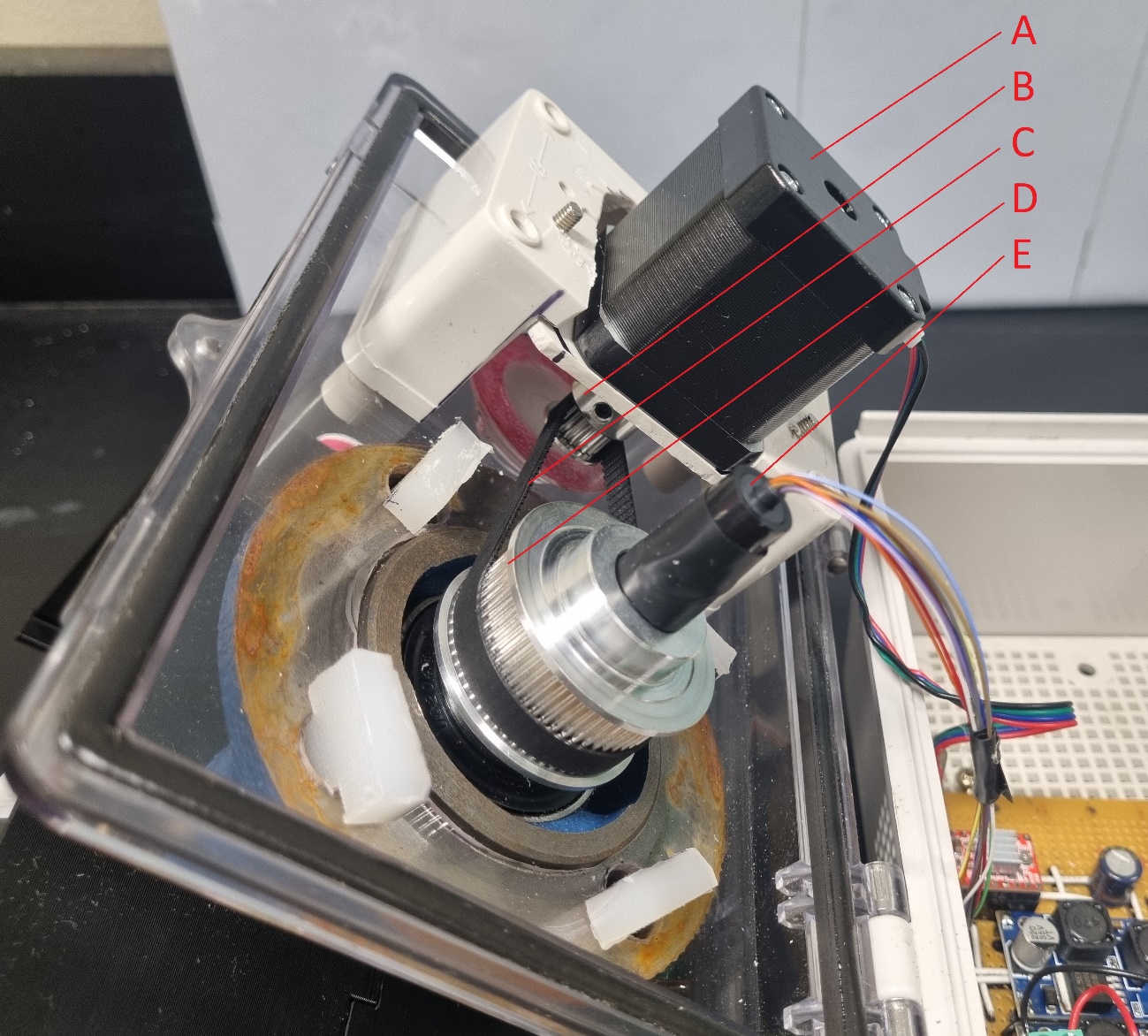


**Code 2**. R code to correct the laser rangefinder offset.

| laser_offset = function(raw, offset, right_side){    #True_dist  raw$true_dist = sqrt(raw$distance^2 + offset^2)    #Tan_radian  raw$tan_radian = offset / raw$distance    #Tan_degrees  raw$tan_degrees = raw$tan_radian * 180/pi    #True_dist (according with the side)  if(right_side){  raw$true_bearing = raw$bearing - raw$tan_degrees  raw$true_bearing[raw$true_bearing < 0 & !is.infinite(raw$true_bearing)] = 360 + raw$true_bearing[raw$true_bearing < 0 & !is.infinite(raw$true_bearing)]  }else{  raw$true_bearing = raw$bearing + raw$tan_degrees  raw$true_bearing[raw$true_bearing > 360 & !is.infinite(raw$true_bearing)] = raw$true_bearing[raw$true_bearing > 360 & !is.infinite(raw$true_bearing)] %% 360  }    # Zero is the default return value when the laser bean reflex is not detected (shot beyond measurement range).  # Since the offset effect is smaller at higher ranges, it become negligible at large ranges.  raw$true_dist[raw$distance == 0] = 0  raw$true_bearing[raw$distance == 0] = raw$bearing[raw$distance == 0]  #save the data  raw$distance = raw$true_dist  raw$bearing = raw$true_bearing    #clean  raw$true_dist = NULL  raw$tan_radian = NULL  raw$tan_degrees = NULL  raw$true_bearing = NULL    return(raw)  } |
| --- |

**Code 3.** Python software to control the LiDAR operation.

| # EcoLiDAR Software  # Version: 2.0 (25/06/2022)  # Written by: Calebe Pereira Mendes  # More details available at:  # Mendes and T-Lim. EcoLiDAR: an Economical LiDAR scanner for Ecological research  # The following code allow the usage of the EcoLiDAR device described in the manuscript above.  # The code was written aiming of maximum readability for python beginners.  # We are aware that this code can be improved in multiple ways. Feel free to edit it as needed.  #########################################################################  ########## INICIALIZATION PROCESS ##########  ### Loading the required libraries  import RPi.GPIO as GPIO # To control the raspberry’s general-purpose input/output (GPIO)  import time # To use time-related functions  import os # To allow communication between the python script and the Raspberry Pi OS  from smbus import SMBus # To connect the LiDAR module to the raspberry with I2C interface.  ### Make functions to connect the Lidar with I2C interface  class LIDARLite_v3hp(): #Create a class object for the Lidar module  def __init__(self):  self.lidar_address = 0x62 # 0x62 is the default I2C register address of the Garmin Lidar-Lite v3hp    def Start(self): # Function to start the I2C connection  try:  # Start  self.bus = SMBus(1)  time.sleep(0.3)    # Configure (using default register addresses as described in the LiDAR datasheet)  self.bus.write_byte_data(self.lidar_address, 0x02, 0x80)  time.sleep(0.3)  self.bus.write_byte_data(self.lidar_address, 0x04, 0x00)  time.sleep(0.3)  self.bus.write_byte_data(self.lidar_address, 0x12, 0x03)  time.sleep(0.3)  self.bus.write_byte_data(self.lidar_address, 0x1c, 0x00)  time.sleep(0.3)    return 1  except:  return -1    def RangeFinder(self): #Create a function to measure the distance using the LiDAR module  self.bus.write_byte_data(self.lidar_address, 0x00, 0x04)  distance_cm = self.bus.read_i2c_block_data(self.lidar_address, 0x8f, 2)  return (distance_cm[0] << 8 \| distance_cm[1])  ### define the GPIOs that will be used.  led_gpio = 14 #LED  Start_button_gpio = 25 #button  Stop_button_gpio = 8 #button  pan_motor_step_gpio = 24 #Pan-axis stepper motor step  tilt_motor_step_gpio = 18 #Tilt-axis stepper motor step  pan_motor_enable_gpio = 23 #Pan axis step motor enable  tilt_motor_enable_gpio = 15 #Tilt axis step motor enable  ms1_gpio = 7 # Microstepping for Pan axis step motor  ms2_gpio = 1 # Microstepping for Pan axis step motor  #ms3_gpio = X # Microstepping for Pan axis step motor (not in current use)  lidarSDA_gpio = 2 #SDA (Blue) pin2  lidarSCL_gpio = 3 #SCL (Green) pin3  ### Define the initial state of the pins  GPIO.setwarnings(False)  GPIO.setmode(GPIO.BCM)  GPIO.setup(led_gpio,GPIO.OUT)  GPIO.setup(Start_button_gpio ,GPIO.IN, pull_up_down=GPIO.PUD_DOWN)  GPIO.setup(Stop_button_gpio,GPIO.IN, pull_up_down=GPIO.PUD_DOWN)  GPIO.setup(pan_motor_step_gpio,GPIO.OUT)  GPIO.setup(tilt_motor_step_gpio,GPIO.OUT)  GPIO.setup(pan_motor_enable_gpio,GPIO.OUT)  GPIO.setup(tilt_motor_enable_gpio,GPIO.OUT)  GPIO.setup(ms1_gpio,GPIO.OUT)  GPIO.setup(ms2_gpio,GPIO.OUT)  #GPIO.setup(ms3_gpio,GPIO.OUT)  ### Define the initial state of the GPIOs  GPIO.output(led_gpio,GPIO.HIGH)  GPIO.output(pan_motor_step_gpio,GPIO.LOW)  GPIO.output(tilt_motor_step_gpio,GPIO.LOW)  GPIO.output(pan_motor_enable_gpio,GPIO.HIGH)  GPIO.output(tilt_motor_enable_gpio,GPIO.HIGH)  GPIO.output(ms1_gpio,GPIO.LOW)  GPIO.output(ms2_gpio,GPIO.LOW)  #GPIO.output(ms3_gpio,GPIO.LOW)  ### Define variables  step_time = 1350 #microseconds.  step_size = 1/4 # Each microstep have 1/4 of a full step. full step = 1. Do not use float!!  bearing = 0 # Bearing in which the scan start (0 = pointing to the north)  inclination = -36 # The inclination where the scan start (0 = horizon, 90 = zenith, -90 = nadir)  full_step_angle = 1.8 # Defined by the step motor model (1.8 for the model 17HS4401)  gear_ratio = 3/1 # Gear ratio for the pan axis step motor  ### Make a function to stop the software (for the stop button)  def CheckStopButton():  if GPIO.input(Stop_button_gpio): #In the case the button is pressed, shine the led and disable the motors  GPIO.output(led_gpio,GPIO.HIGH)  GPIO.output(pan_motor_enable_gpio,GPIO.HIGH)  GPIO.output(tilt_motor_enable_gpio,GPIO.HIGH)  new_scam.write("Warning: Scan stopped") #leave a warning in the saved file  new_scam.close()  while True: #locks the software in an idle state  time.sleep(1)  ### Make a function to advance to the next pan step/microstep  def Pan_step():  # Send an electric sign for the motor driver  GPIO.output(pan_motor_step_gpio,GPIO.HIGH)  time.sleep(5e-06)  GPIO.output(pan_motor_step_gpio,GPIO.LOW)    # Update the bearing value  global bearing  global step_size  global full_step_angle  global gear_ratio    bearing = bearing + (full_step_angle / gear_ratio) * step_size    # If a full circumference is complete, advance a tilt step  if bearing >= 360:  bearing = bearing%360  Tilt_step()    ### Make a function to advance to the next tilt step/microstep  def Tilt_step():  # Send an electric sign for the motor driver  GPIO.output(tilt_motor_step_gpio,GPIO.HIGH)  time.sleep(5e-06)  GPIO.output(tilt_motor_step_gpio,GPIO.LOW)    # Update the bearing value  global inclination    inclination = inclination + full_step_angle * 1/4 # The tilt motor microstepping is 1/4        ### Make a function for changing the step size of the Pan Axis Motor (ms3 is not currently used)  def Change_step(step):  global step_size  if step == 1:  GPIO.output(ms1_gpio,GPIO.LOW)  GPIO.output(ms2_gpio,GPIO.LOW)  #GPIO.output(ms3_gpio,GPIO.LOW)  step_size = step  elif step == 1/2:  GPIO.output(ms1_gpio,GPIO.HIGH)  GPIO.output(ms2_gpio,GPIO.LOW)  #GPIO.output(ms3_gpio,GPIO.LOW)  step_size = step  elif step == 1/4:  GPIO.output(ms1_gpio,GPIO.LOW)  GPIO.output(ms2_gpio,GPIO.HIGH)  #GPIO.output(ms3_gpio,GPIO.LOW)  step_size = step  elif step == 1/8:  GPIO.output(ms1_gpio,GPIO.HIGH)  GPIO.output(ms2_gpio,GPIO.HIGH)  #GPIO.output(ms3_gpio,GPIO.LOW)  step_size = step  elif step == 1/16:  GPIO.output(ms1_gpio,GPIO.HIGH)  GPIO.output(ms2_gpio,GPIO.HIGH)  #GPIO.output(ms3_gpio,GPIO.HIGH)  step_size = step    ### Set the folder where the scans will be saved  os.chdir("/home/pi/Desktop/Lidar_scans")  ### Initiate the LiDAR module    lidar = LIDARLite_v3hp()  connect = lidar.Start()  if connect < 0: # in case of communication failure  GPIO.output(led_gpio,GPIO.HIGH)  GPIO.output(pan_motor_enable_gpio,GPIO.HIGH)  GPIO.output(tilt_motor_enable_gpio,GPIO.HIGH)  while True: #locks the software in an idle state  time.sleep(1)  ########## WAITING FOR THE START BUTTON ##########  chronometer = time.time()  wait_for_start = True  while wait_for_start:    if GPIO.input(Start_button_gpio ):  wait_for_start = False    CheckStopButton()    # Blink the led, The number of blinks refer to the name of the new scan file.  # example: 1 blink = “scan_1.txt”, 5 blinks = “scan_5.txt”, etc.  if (time.time() - chronometer) > 5:  for i in range(0,len(os.listdir())+1):  GPIO.output(led_gpio,GPIO.HIGH)  time.sleep(0.3)  GPIO.output(led_gpio,GPIO.LOW)  time.sleep(0.3)  chronometer = time.time()    ########## SCANNING ##########  ### Create the scan files  new_scam_name = "scan_%s.csv" %str(len(os.listdir())+1)  new_scam = open(new_scam_name, "w+")  time.sleep(0.5)  new_scam.write("bearing,inclination,distance,time\n")  ### Wait for 30 seconds to allow the operator to get distance  chronometer = time.time()  while time.time() < chronometer + 30: #30 sec  GPIO.output(led_gpio,GPIO.HIGH)  time.sleep(0.1) #wait 5 seconds  GPIO.output(led_gpio,GPIO.LOW)  time.sleep(0.1) #wait 5 seconds    CheckStopButton()  distance = lidar.RangeFinder() #prepare the LiDAR buffer for the next measurement  time.sleep(0.2)  #Enable the motors  GPIO.output(pan_motor_enable_gpio,GPIO.LOW)  GPIO.output(tilt_motor_enable_gpio,GPIO.LOW)  time.sleep(1)  Scanning = True  ### Start the rotation with higher torque  Change_step(1)  for i in range(0,300): # begin with half revolution at step 1 (300 steps)  time.sleep((step_time*6)/1000000)    GPIO.output(pan_motor_step_gpio,GPIO.HIGH)  time.sleep(5e-06)  GPIO.output(pan_motor_step_gpio,GPIO.LOW)  # Change for a smoother rotation  Change_step(1/4) # change to half revolution at 1/4 microstepping (1200 microsteps)  for i in range(0,1200):  time.sleep(step_time/1000000)    GPIO.output(pan_motor_step_gpio,GPIO.HIGH)  time.sleep(5e-06)  GPIO.output(pan_motor_step_gpio,GPIO.LOW)    ### Scanning loop    chronometer = time.time()  CheckStopButton()  distance = lidar.RangeFinder()  _ = new_scam.write("%s,%s,%s,%s\n" %(bearing,inclination,distance,time.time()*1000))    Pan_step()  while chronometer+(step_time/1000000) > time.time():  pass  ### Saving the data  new_scam.close()  # Turning off the motors  GPIO.output(pan_motor_enable_gpio,GPIO.HIGH)  GPIO.output(tilt_motor_enable_gpio,GPIO.HIGH) |
| --- |

**Manual 4.**

**User Manual**

This manual describes the operation of the EcoLiDAR. Note that changes in the software or building process may require changes to the operation. Therefore, this manual only applies for a device built exactly as described in the manuscript, using the provided software.

Scanning

1. Connect the power cable to the battery and to the power connector in the stationary base. Turn on the power switch. Note that during the OS booting process, the rotation of the head is blocked by the stepper motors. Do not force the rotating head or it can damage the gimbal or the motor drivers. The rotating head can be rotated freely after the booting.
2. When the operational system completes the initialization, the LED located in the rotating head electronic board will start to blink. The number of blinks (half second each) indicate the name of the new scan file that will be create. The name is: “scan_X.csv”, where “X” is the number of blinks (e.g. 1 blink = “scan_1.csv”, 5 blinks = “scan_5.csv”, etc.).
3. Before starting the scan, attach the LiDAR scanner to a tripod. Level the device using a round bubble level. Point the laser rangefinder to its initial position (bearing 0, tilt angle -40°). Note that “bearing 0” means North, while “tilt angle -40°” means 40° below the horizon.
4. To initiate the scan, press the start button. The button need to be pressed during the 5s interval between the LED blinks, otherwise it will not be detected. After pressing the start button, the LED will start to blink quickly during 30 seconds, giving time for the operator to get distance to the lidar device (to not interfere in the scan). After that, the scan will begin. A complete scan takes from ~20 minutes to several hours, according to the resolution used.
5. The scan can be cancelled at any time by pressing the cancel button. After detecting the cancel button being pressed, the LiDAR device enters in a “inactive mode”. To leave the inactive mode, the LiDAR device need to be restarted using the power swich.
6. In order to retrieve the scanned data files or to change the scan settings it is needed to access the EcoLiDAR desktop. The scan settings can be adjusted by changing the parameters in the EcoLiDAR Software.

Connecting to the EcoLiDAR desktop.

Since the EcoLiDAR is based on a Raspberry Pi Zero running Linux, it is possible to access its desktop to retrieve the scan files or to change the scan settings. There are two ways to access the desktop. The wireless connection have the advantage of being easier to use and available on off grid conditions, but it need to be configured first.

Wired connection:

1. The wired connection consists in disconnecting the Raspberry Pi Zero from the LiDAR device and connecting it to a monitor, keyboard, mouse and USB power source. Since the Raspberry pi zero is a computer by itself, the files can be accessed just like in any Linux computer. Note that there is no need to physically remove the Raspberry from the LiDAR, as long as the connections are accessible and the power wires between the Raspberry and the 5V voltage regulator are disconnected (to avoid reverse current on the LiDAR voltage regulators). Indeed, a good way to debug the EcoLiDAR is to power the Raspberry by USB while connected to the LiDAR device (except the power wires connecting it to the 5v voltage regulator).

Configuring the Wireless connection:

1. To enable to wireless connection, first, connect to the EcoLiDAR desktop using the wired connection (this step can be performed during the setup of the EcoLiDAR software).
2. Enable the wi-fi on the Raspberry Pi Zero.
3. Use any device (such as a smartphone or computer) to create a wi-fi hotspot. Record the wi-fi name and password, for they will be needed later.
4. Connect the Raspberry to the wi-fi hotspot and select the option to connect automatically to the network. This way, every time the EcoLiDAR detect a wi-fi network with the same name and password, it will connect to it.
5. Install a remote access software of choice in the Raspberry Pi Zero. VNC Viewer is a good option.

Using the Wireless connection:

1. Turn on the Eco-LiDAR.
2. Use any device to create a wi-fi hotspot using the network name and password defined in the configuration process. The EcoLiDAR should connect automatically to the network.
3. Use the remote access software of choice to access the EcoLiDAR desktop through the wi-fi hotpot connection.

**Fig 5.** Laser alignment process. The infrared (IR) laser emitted by the rangefinder can be visualized using low-light cameras, webcams or smartphones. This way, the laser orientation in relation to the rotating box can be measured, aligned, and added to the software to produce precise scans. On the left, the IR laser incidence on a rectangular target surface was captured using a video camera. On the right, a line is drawn to point the laser location.

**
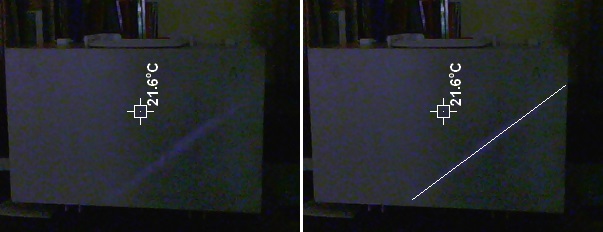
**

**Fig 6.** Tilt angle stopper. When the laser rangefinder reaches its starting position, the tilt angle stopper (left) collides with the rotating head`s main box (right).


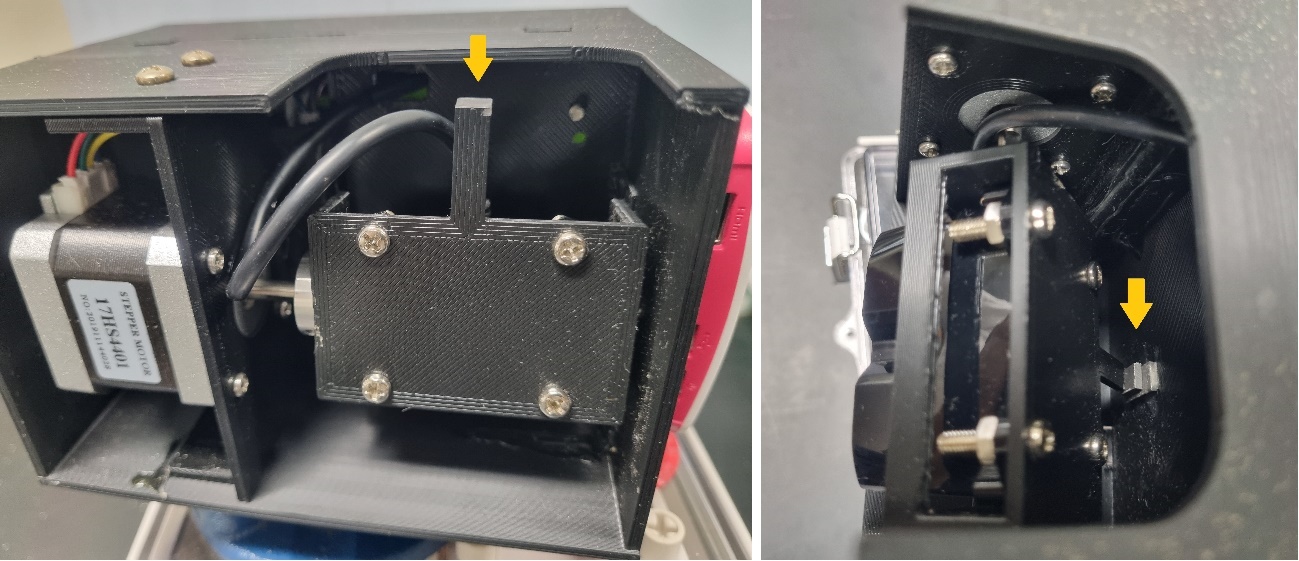


**Table 7.** Tree height measurements. Comparison between tree height measurements in field and height estimation based on the EcoLiDAR produced point cloud. All measurements are in meters.

| Tree ID | Height (at field) | Height estimation (EcoLIDAR point cloud) | Error |
| --- | --- | --- | --- |
| 1 | 19.6 | 19.7 | 0.1 |
| 2 | 4.4 | 4.38 | -0.02 |
| 3 | 5 | 5.14 | 0.14 |
| 4 | 21.8 | 19.97 | -1.83 |
| 5 | 16.8 | 17.93 | 1.13 |
| 6 | 18.6 | 19 | 0.4 |
| 7 | 18.2 | 16.56 | -1.64 |

**Table 8.** Price table of the LiDAR components (in USD).

| **Main components** | **Amount** | **Price (USD)** | **Subtotal** |
| --- | --- | --- | --- |
| Garmin LIDAR Lite V3HP | 1 | 160 | 160 |
| Raspberry Pi Zero | 1 | 10.95 | 10.95 |
| NEMA 17 bipolar stepper motor (17HS4401) | 2 | 10.20 | 20.40 |
| A4988 stepper motor driver module | 2 | 1.60 | 3.20 |
| XL6009 4A step-up module | 2 | 0.90 | 1.80 |
| LM2596 3A step-down module | 1 | 0.75 | 0.75 |
| Cooling fan (40x40x10 mm, 5V) | 1 | 1.25 | 1.25 |
| Battery (12V, 7Ah) | 1 | 23.40 | 23.40 |
| SD card (16GB +) | 1 | 10.95 | 10.95 |
| **Case** |  |  |  |
| Electric juction box | 1 | 29.20 | 29.20 |
| Case for Raspberry pi zero | 1 | 7.30 | 7.30 |
| 3D print filament (1kg spool) | 1 | 22 | 22 |
| **Two-axis gimbal** |  |  |  |
| GT2 timing pulley (60 Teeth, 20mm bore) | 1 | 6.6 | 6.6 |
| GT2 timing pulley (20 Teeth, 5mm bore) | 1 | 1.10 | 1.10 |
| GT2 timing belt (200mm, 6mm wide) | 1 | 2.35 | 2.35 |
| Flanged bearing (20mm bore) | 1 | 11 | 11 |
| Slip ring (10 wires) | 1 | 27 | 27 |
| 5mm aluminium key hub | 1 | 7.30 | 7.30 |
| **Electronic board** |  |  |  |
| Bread board | 1 | 1.10 | 1.10 |
| 100μF Electrolytic Capacitor 50V DC | 2 | 0.50 | 1 |
| 680μF Electrolytic Capacitor 6.3V DC | 1 | 0.60 | 0.60 |
| 220 ohms resistor | 1 | 0.08 | 0.08 |
| 10K ohms resistor | 2 | 0.08 | 0.16 |
| LED | 1 | 0.15 | 0.15 |
| Header 1x40 Ways (female) | 2 | 0.35 | 0.70 |
| Push button | 2 | 0.70 | 1.40 |
| Power switch | 1 | 0.70 | 0.70 |
| Crocodile clip conector | 2 | 0.70 | 1.40 |
| 5.5x2.1mm DC Jack (female) | 1 | 0.70 | 0.70 |
| 5.5x2.1mm DC Jack (male) | 1 | 0.70 | 0.70 |
| Water level | 1 | 2.20 | 2.20 |
| Jumpers (pack) | 1 | 2.20 | 2.20 |
| Screws and bolts (multiple sizes) | 1 | 7.30 | 7.30 |
| AWG 22 electric wires (red, black, 3rd color) | 3 | 3.65 | 10.95 |
| **TOTAL** |  |  | **377.89** |

**Fig 9. EcoLiDAR deployed for test. Pictures of the EcoLiDAR scanner deployed in the location which produced the scan displayed in the manuscript Fig. 5.**

**
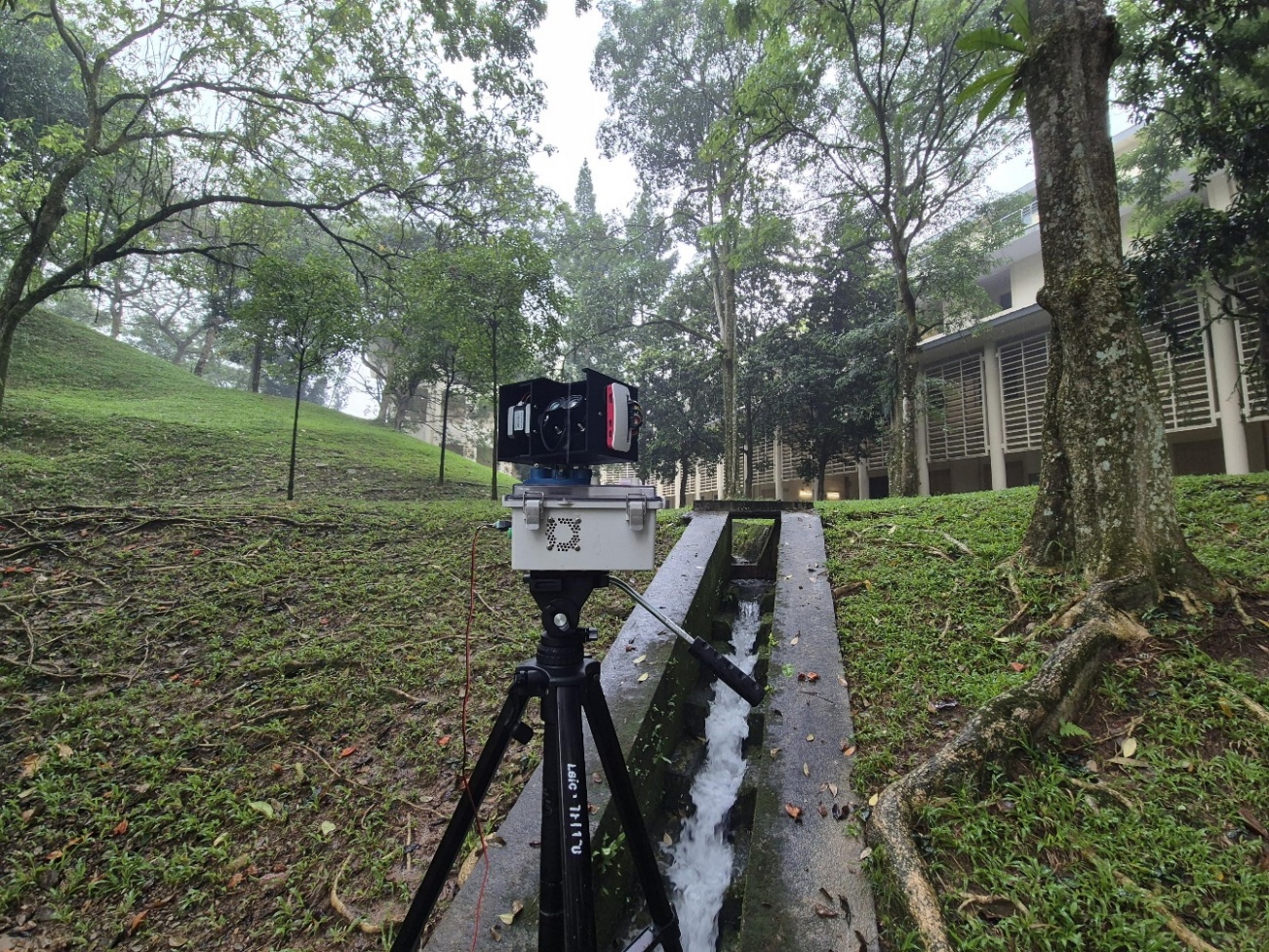

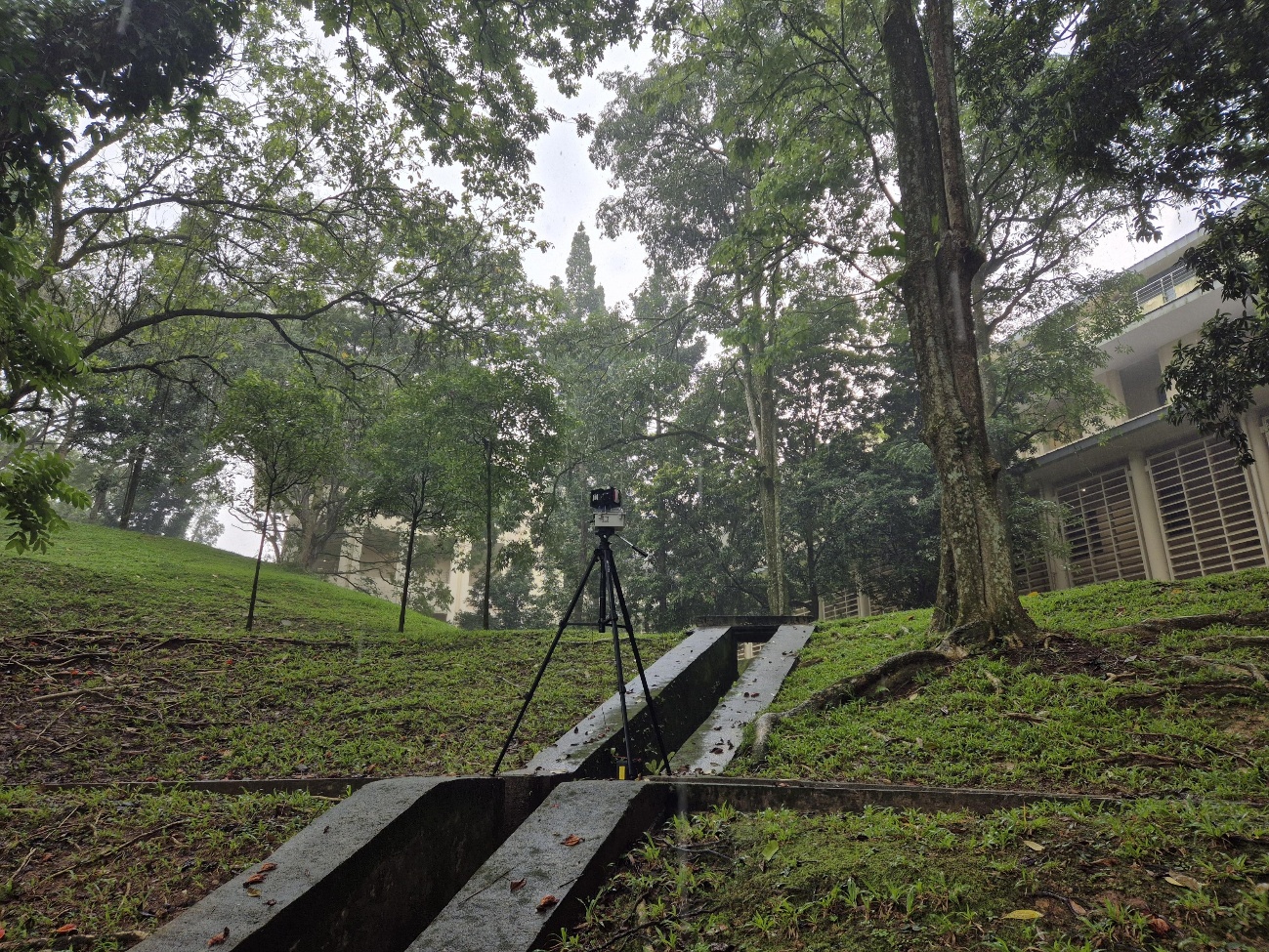
**
