# Supplementary figures and images for: EcoLiDAR: An economical LiDAR scanner for ecological research
Source: PLoS One. 2024 Jun 25;19(6):e0298712. doi: 10.1371/journal.pone.0298712 (PMC11198765; doi:10.1371/journal.pone.0298712)

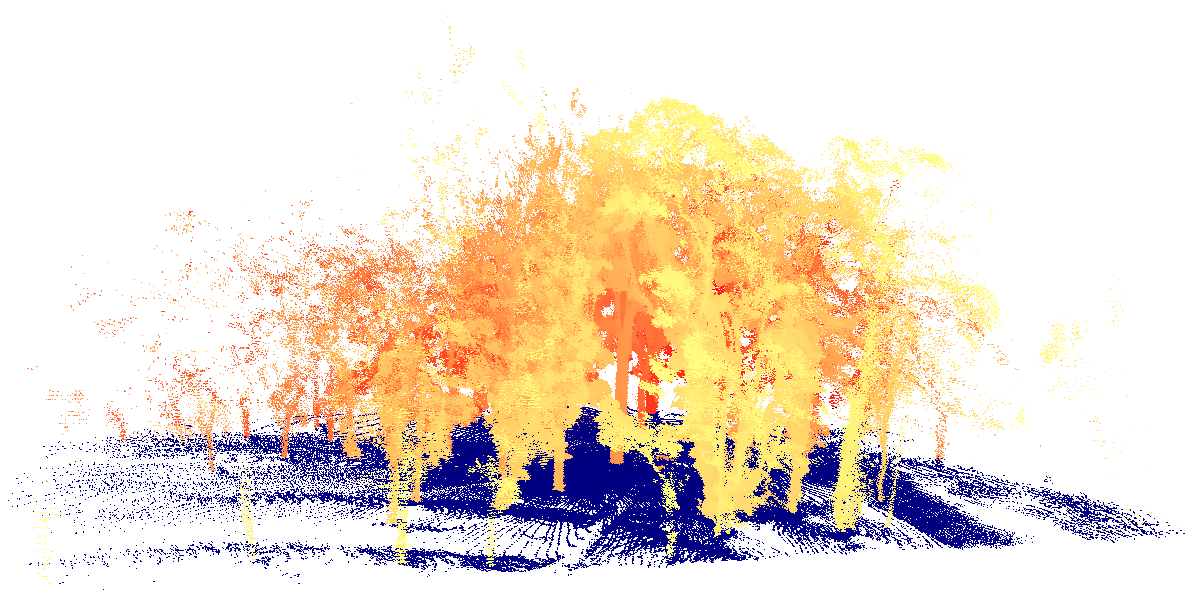

Supplement: S2 File — Raw and processed EcoLiDAR scans, with the respective analysis R codes. (ZIP) [file pone.0298712.s002.zip › EcoLiDAR-main/Merged point cloud - normalized.png]

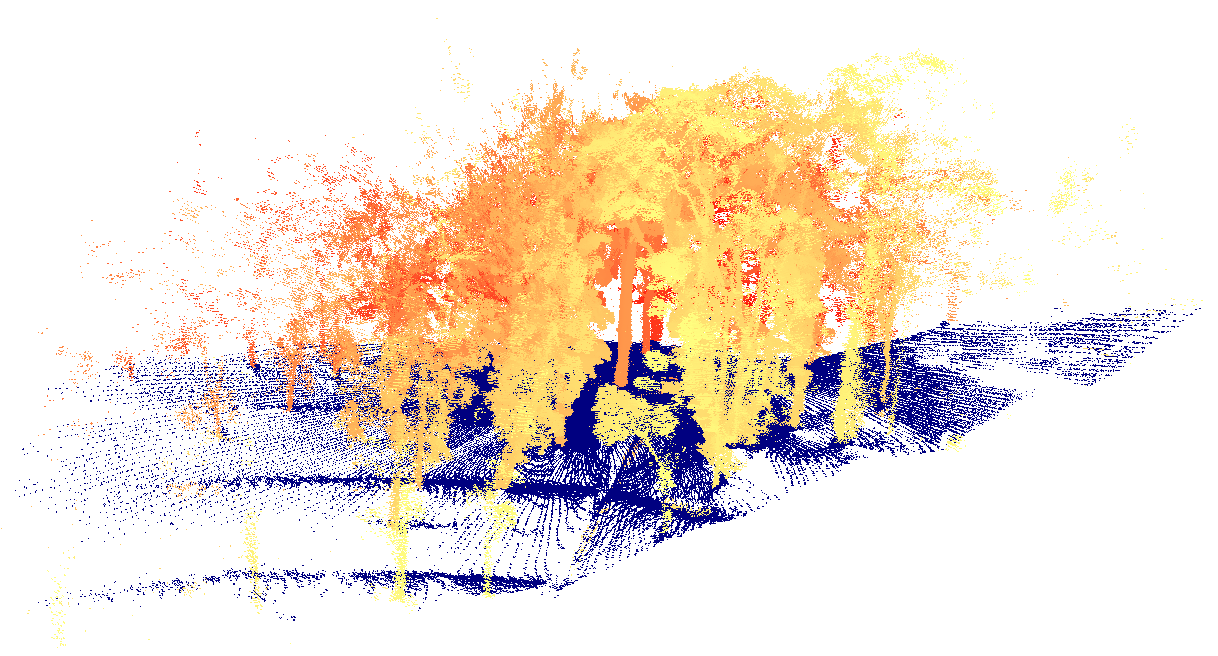

Supplement: S2 File — Raw and processed EcoLiDAR scans, with the respective analysis R codes. (ZIP) [file pone.0298712.s002.zip › EcoLiDAR-main/Merged point cloud.png]

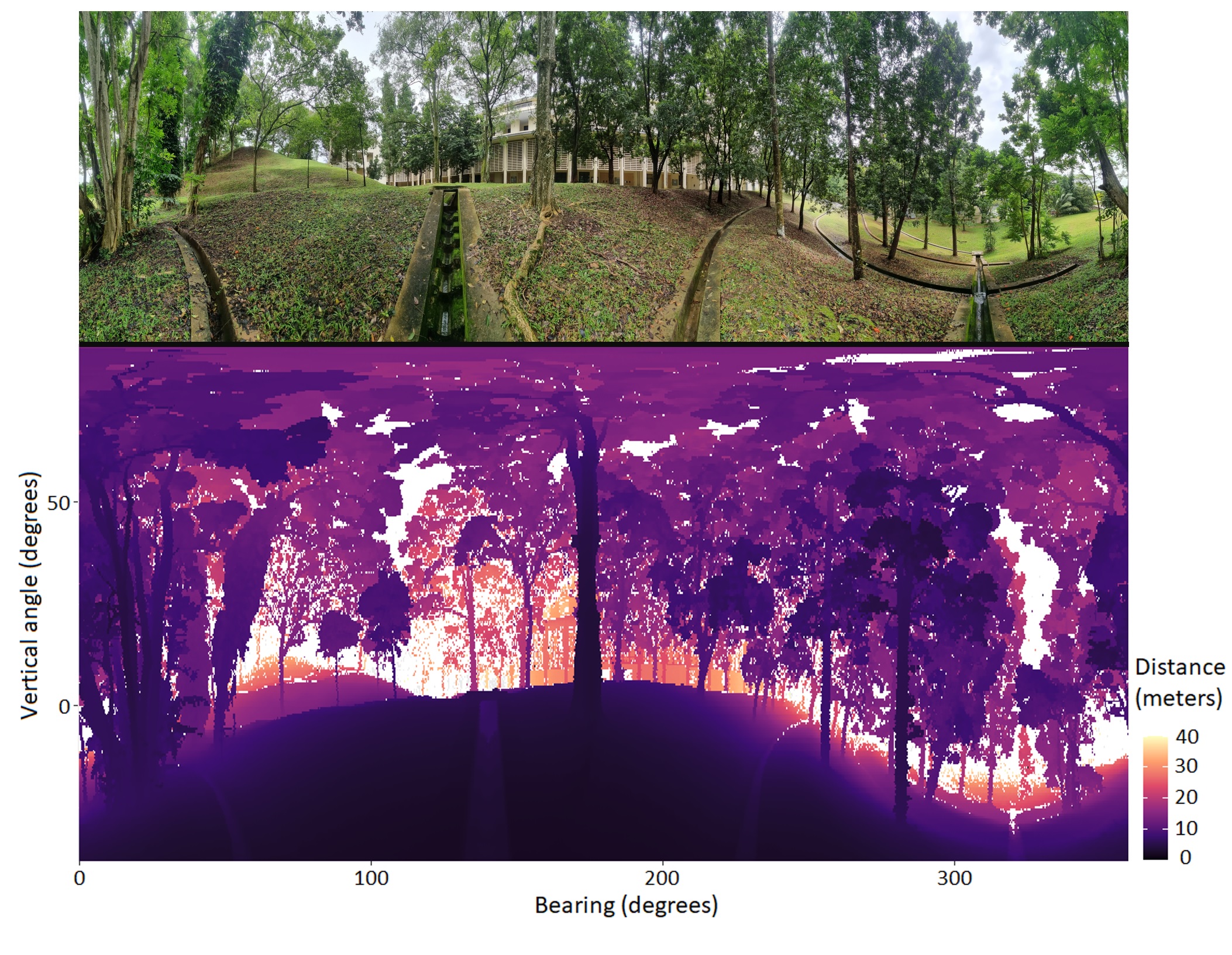

Supplement: S2 File — Raw and processed EcoLiDAR scans, with the respective analysis R codes. (ZIP) [file pone.0298712.s002.zip › EcoLiDAR-main/Panoramic_merged.jpg]

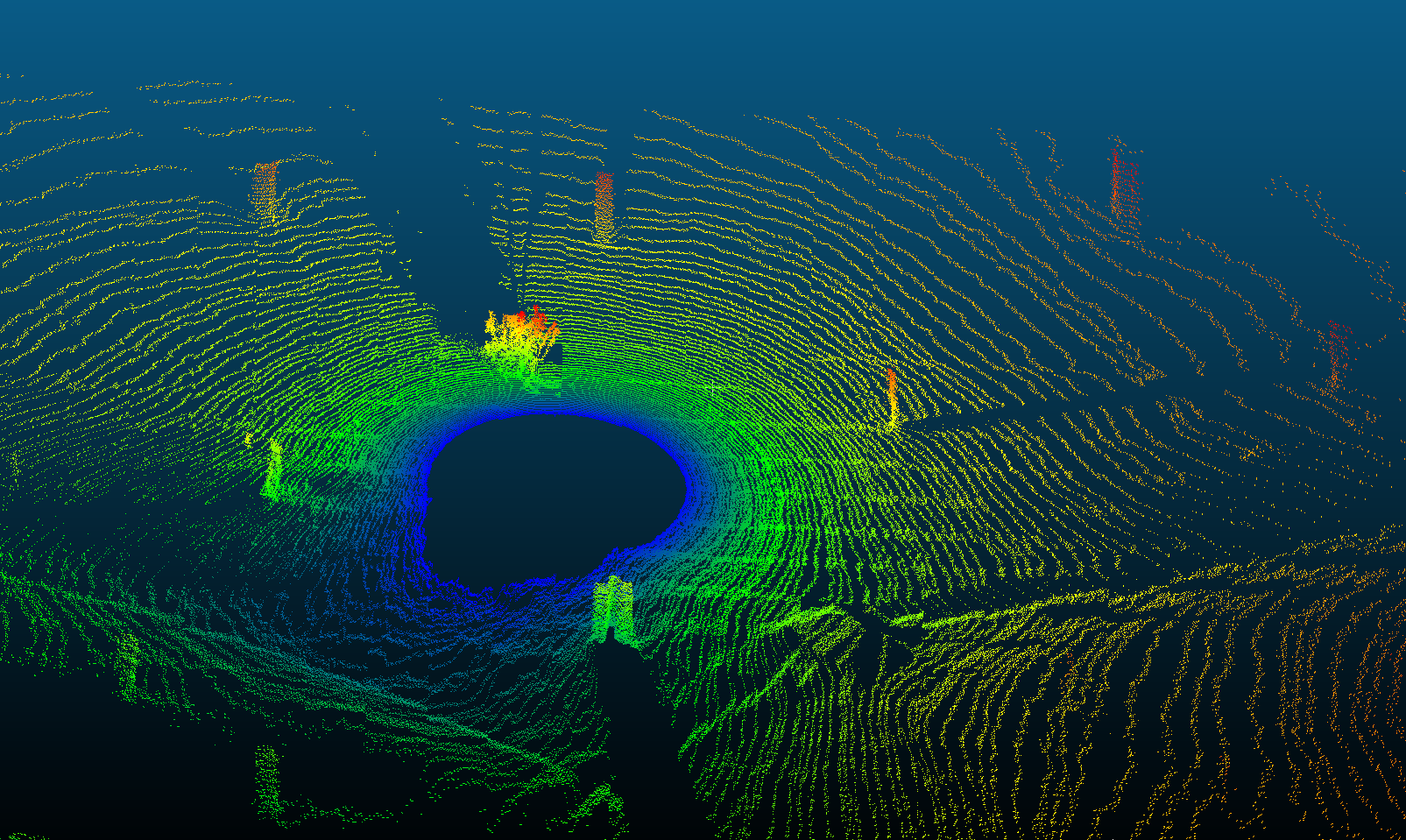

Supplement: S2 File — Raw and processed EcoLiDAR scans, with the respective analysis R codes. (ZIP) [file pone.0298712.s002.zip › EcoLiDAR-main/Tree trunk cross section at breast height.png]
